# Preparation of a High-Silicon ZSM-5 Molecular Sieve Using Only Coal Gangue as the Silicon and Aluminum Sources

**DOI:** 10.3390/ma16124338

**Published:** 2023-06-12

**Authors:** Yunsheng Zheng, Junxia Zhou, Zhijun Ma, Xingyuan Weng, Liang Cheng, Guorong Tang

**Affiliations:** 1 College of Mining, Liaoning Technical University, Fuxin 123000, China; zys_lgd@126.com (Y.Z.); wengxingyuan2008@163.com (X.W.); chengliang9506@126.com (L.C.); 15739958369@163.com (G.T.); 2 College of Architecture and Transportation, Liaoning Technical University, Fuxin 123000, China; zhoujunxia@lntu.edu.cn

**Keywords:** coal gangue, high-value utilization, pressure acid leaching, ZSM-5 molecular sieve

## Abstract

The traditional preparation of ZSM-5 molecular sieves relies on chemical reagents to provide silicon and aluminum sources, which are limited as raw materials and cannot be commonly used in production practice. Using coal gangue as the raw material and using medium-temperature chlorination roasting and the pressure acid leaching process to control the silicon–aluminum ratio [n(Si/Al)] of coal gangue, a ZSM-5 molecular sieve was prepared using the alkali melting hydrothermal method. The pressure acid leaching process solved the limitation that kaolinite and mica cannot simultaneously be activated. Under optimal conditions, the n(Si/Al) of the coal gangue increased from 6.23 to 26.14 and complied with the requirements for the synthesis n(Si/Al) of a ZSM-5 molecular sieve. The effect of n(Si/Al) on the preparation of the ZSM-5 molecular sieve was studied. Finally, spherical granular ZSM-5 molecular sieve material with a microporous specific surface area of 169.6329 m^2^/g, an average pore diameter of 0.6285 nm, and a pore volume of 0.0988 cm^3^/g was prepared. Providing ideas for the high-value utilization of coal gangue, it is significant for solving the problem of coal gangue solid waste, as well as the problem of ZSM-5 molecular sieve feed stock.

## 1. Introduction

A ZSM-5 molecular sieve is a widely used microporous substance. Due to its excellent properties, it has been studied by many experts [1,2,3,4]. The special framework structure of the ZSM-5 molecular sieve provides it with unique shape selectivity, thermal stability, and acid resistance [5,6,7]. It is widely used in the field of traditional chemical catalysis [8,9,10,11,12]. ZSM-5 molecular sieves have been modified, showing good durability in catalytic cracking reactions using n-hexane and isopropyl-benzene [13,14,15] and excellent selectivity in the reaction of methanol to aromatics, gasoline, and olefin [16,17]. In the alkylation reaction, a ZSM-5 molecular sieve was modified by Pt or combined with CuO/ZnO/Al_2_O_3_ to produce toluene and xylene [18,19,20]. In the isomerization reaction, the ZSM-5 molecular sieve had a certain acidity, and ZSM-5 with suitable acidity was prepared by combined treatment with alkali vapor that demonstrated an effective catalytic performance for the aromatization and isomerization of ethylene [21,22]. Fe can also be introduced into the ZSM-5 molecular sieve framework to control its acidity, which helps to reduce coking of the xylene isomerization reaction, enhancing the stability of catalysts [23]. In the field of new applications, ZSM-5 molecular sieves can be used as an environmental protection material. A ZSM-5 molecular sieve composited with silica was shown to have an excellent adsorption capacity for organic pollutants and compared with the Z-Scheme g-C_3_N_4_/Fe_3_O_4_/Ag_3_PO_4_@Sep magnetic nanocomposite photocatalytic material prepared using the coprecipitation method, both of them have good repeatability utilization performance [24,25]. In addition, ZSM-5 modified with nitrogen has excellent adsorption capacity for benzene [26]. In electrochemistry, a ZSM-5 molecular-sieve-modified electrode was obtained by mixing ZSM-5 with methyl acrylate on a glassy carbon electrode [27]. An electrode material prepared using CO_2_-activated zsm-5 showed good reversibility and a stable long-term cycle life during repeated charging and discharge cycles [28]. Xi et al. [29] investigated PEO-LiClO_4_-ZSM5 composite electrolytes. This is significant in the field of all-solid-state lithium-ion secondary batteries due to the formation of conductive channels on the surface of ZSM-5 that facilitate the migration of Li ions, resulting in increased ionic conductivity of the electrolyte. Regarding material anticorrosion, a ZSM-5 molecular sieve added to waterborne coatings could improve the corrosion resistance of the coatings; this is expected to become an environmentally friendly corrosion-resistant material to replace traditional coatings [30]. Preparation methods include the hydrothermal system synthesis method [31,32], non-aqueous system synthesis method [33,34,35], solid-phase synthesis method [36,37], and dry adhesive system synthesis method [38,39]. Sungtak et al. [40] used microwave-assisted heating to synthesize mesoporous ZSM-5 molecular sieves with larger and wider grain sizes. Ghassan et al. [41] successfully prepared ZSM-5 molecular sieves with a grain size of 52.2 nm, a crystallinity of 103%, and a surface area of 325.14 m^2^/g by controlling the crystallization conditions.

The traditional preparation of ZSM-5 molecular sieves mainly uses chemical reagents as raw materials, but this increases the preparation costs due to material constraints. Therefore, chemical reagents cannot be widely used in production practice. Ye et al. [42] successfully synthesized a ZSM-5 zeolite catalyst with tailings as the raw material using a template-free method. Coal gangue mainly contains SiO_2_ and Al_2_O_3_; they account for approximately 50–80% of the coal gangue component [43], making it an appropriate raw material for the synthesis of ZSM-5 molecular sieves. Although there has been progress in the preparation of ZSM-5 molecular sieves from coal gangue in recent years, shortcomings remain. On the one hand, because the n(Si/Al) of a ZSM-5 molecular sieve is required to be above 15 [44], and because the n(Si/Al) of coal gangue itself is relatively low, it is necessary to internally add a silicon source during the preparation process to improve the n(Si/Al) of the crystallization system. Jin et al. [45] successfully prepared a ZSM-5 molecular sieve via hydrothermal synthesis using coal gangue as the raw material, adding silicon sources to improve the n(Si/Al) of the synthesis system. This method did not completely overcome the limitations of preparing ZSM-5 molecular sieves using chemical reagents, such as silicon and aluminum sources, and only reduced the number of chemical reagents used. On the other hand, ZSM-5 molecular sieves have been prepared using a coal gangue alkaline leaching supernatant. Li et al. [46] prepared porous ZSM-5 molecular sieves with a coal gangue alkaline leaching supernatant as the raw material using hydrothermal synthesis, TPABr as a microporous template, and CTAB as a mesoporous template. This method rendered several of the silicon and aluminum components unusable in the precipitation, reducing the utilization efficiency of the coal gangue. There have been few reports on the preparation of ZSM-5 molecular sieves using only coal gangue as a silicon–aluminum source.

Therefore, aiming at the problem of the comprehensive utilization of coal gangue solid waste, and raw materials for the synthesis of ZSM-5 molecular sieves, in this study, we used only coal gangue as the silicon and aluminum source to prepare a ZSM-5 molecular sieve using a two-step method. First, medium-temperature chlorination roasting and pressure acid leaching were used to regulate the n(Si/Al) of the coal gangue. Second, ZSM-5 molecular sieves were prepared via hydrothermal synthesis using ZSM-5 crystal seeds and TPABr as dual-structure directing agents. The effect of n(Si/Al) on the preparation of ZSM-5 molecular sieves from coal gangue was then studied. Our aim was to provide technical and theoretical support for the high-value utilization of coal gangue.

## 2. Materials and Methods

### 2.1. Material Preparation

#### 2.1.1. Control of n(Si/Al) of Coal Gangue via Medium-Temperature Chlorinated Roasting and Pressurized Acid Leaching

To begin, 12 g of coal gangue (CG) (φ = 0.074 mm; *w*t. (SiO_2_) = 46.19%; *w*t. (Al_2_O_3_) = 12.60%; *wt.* (Fe_2_O_3_) = 7.12%) and 1.8 g of ammonium chloride (NH_4_Cl; AR) were evenly mixed, placed in a box-type electric furnace for chlorination, and roasted at 600-900 °C for 2 h. The sample was removed from the furnace and naturally cooled to room temperature; thus, a calcined sample was obtained. We add 2–10 mol/L of hydrochloric acid solution (HCl; AR; 36–38%) to the calcined sample at a solid–liquid ratio of 1:10. We then stirred it evenly, placed it in a high-pressure reactor, immersed it in a drying oven at 120–200 °C under self-generated pressure for 2–10 h, and then allowed it to naturally cool to room temperature. It was then washed with distilled water (self-made in the laboratory) to neutral, and dried to obtain an acid-leached sample. GB/T 1574-2007 was used to analyze the content of SiO_2_ (silico-molybdenum blue spectrophotometry), Al_2_O_3_ (fluorinated EDTA complexometric titration), and Fe_2_O_3_ (ferrotitanium spectrophotometry), and the n(Si/Al) was calculated (Equation (1)). By adjusting the parameters of the roasting temperature, acid salt concentration, and acid leaching time, the n(Si/Al) of the coal gangue could be controlled.

(1) $$n(Si/Al)=\frac{{wt}_{{SiO}_{2}}\times M_{{Al}_{2}O_{3}}}{{wt}_{{Al}_{2}O_{3}}\times M_{{SiO}_{2}}}$$

#### 2.1.2. Hydrothermal Synthesis of the ZSM-5 Molecular Sieve

We mixed 2 g of the acid-leached sample with different amounts of n(Si/Al) and mixed it with 3.2 g sodium hydroxide (NaOH; AR). We placed the sample in a box-type electric furnace for alkali melting at 800 °C for 2 h, then allowed it to naturally cool to room temperature to obtain an alkali melt sample. Based on the molar content of SiO_2_ in the alkali melt sample. The molar content of silicon was taken as unit 1, according to the ratio SiO_2_:xAl_2_O_3_:0.04TPABr:140H_2_O, and mixed evenly with cetyltrimethylammonium bromide (TPABr; AR) and distilled water, and 0.018 g of seed crystals (ZSM-5; AR), and the pH was adjusted to 10.5 by ammonia to obtain the reaction precursor. The sample was transferred to a high-pressure reactor and allowed to stand at 45 °C for 12 h. It was then heated up to 180 °C for hydrothermal crystallization for 24 h and cooled to room temperature; it was then washed, filtered, and dried to obtain the crystallization product. The crystallization product was calcined to remove the template, and ZSM-5 molecular sieves (Z-1, Z-2, Z-3, and Z-4) were obtained.

### 2.2. Material Characterization

An X-ray diffractometer (XRD) (D8 ADVANCE, Bruker, Billerica, MA, USA) was used to perform the phase retrieval analysis of the sample under the following test conditions: Cu targeted K α-Ray, 1.5406 Å wavelength, 40 kV working voltage, 40 mA tube current, 0.05 s step length, and 5–50° scanning range. We calculated the relative crystallinity of the ZSM-5 molecular sieve by comparing and analyzing the crystal structure of the samples with a standard card; Fourier-transform infrared spectroscopy (FT-IR) (Tensor II, Bruker, Billerica, MA, USA) was used to analyze the molecular chemical bonds and the skeleton structure of the ZSM-5 molecular sieve sample. The specific surface area, pore-size distribution, and pore volume of the sample were analyzed using a fully automated volumetric vapor adsorption instrument (3H-2000PMV, BEST). The degassing conditions were a temperature of 573.15 K and a time of 4 h. The specific surface area data of the micropores were obtained via the T-Plot method, and the pore diameter and volume of the micropores were obtained via the H-K method. The surface morphology of the sample was observed using scanning electron microscopy (SEM) (Sigma, St. Louis, MO, USA, ZEISS).

## 3. Results and Discussion

### 3.1. Effect of Acid Leaching Process Conditions on the n(Si/Al) of Coal Gangue

**Figure 1:**
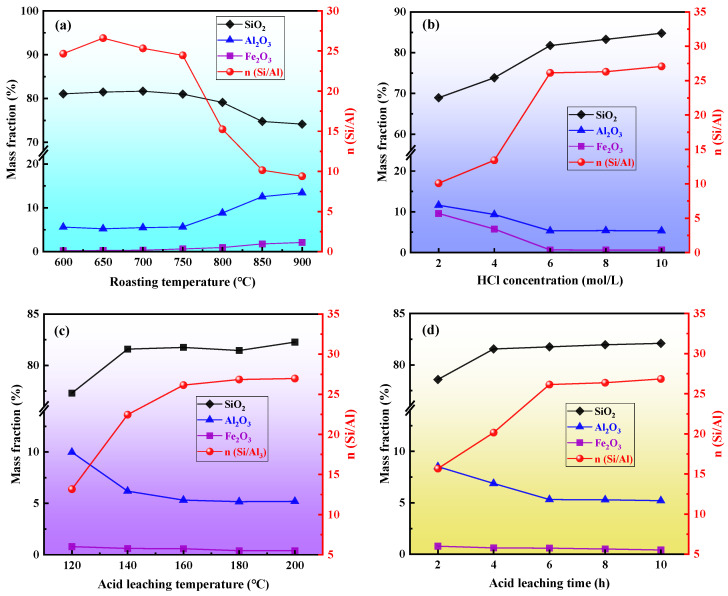
Effect of chlorination roasting and pressurized acid leaching on n(Si/Al) of coal gangue: (**a**) roasting temperature; (**b**) HCl concentration; (**c**) acid leaching temperature; (**d**) acid leaching time.

Due to the low original n(Si/Al) of coal gangue, it cannot meet the requirements for the synthesis of ZSM-5 molecular sieves [44]. Secondly, coal gangue contains Fe and other impure elements. Therefore, it is necessary to regulate the n(Si/Al) and remove the impure elements. Medium-temperature chlorination roasting and the pressure acid leaching process were used in this study. The effects of the roasting temperature, concentration of acid salts, leaching temperature, and leaching time on the n(Si/Al) of the acid-soaked samples are shown in Figure 1a–d.

Figure 1a reflects the effect of roasting temperature on n(Si/Al) under the condition of a hydrochloric acid concentration of 8 mol/L, an acid leaching temperature of 160 °C, and an acid leaching time of 6 h. The analysis in Figure 1a shows that with an increase in roasting temperature, the n(Si/Al) of the acid slag first increased and then decreased. When the calcination temperature was 650 °C, the SiO_2_ content increased from 46.19% to 81.49%, and the Al_2_O_3_ content decreased from 12.60% to 5.20%. This was due to the incomplete conversion of kaolinite to metakaolin at 600 °C. An increase in the calcination temperature helped to transform kaolinite into metakaolin, resulting in greater activation effects and an increase in the n(Si/Al) of the acid-leached samples. When the temperature continued to rise, the n(Si/Al) of the acid-leached slag showed a downward trend. This was due to the metakaolin further decomposing to form amorphous SiO_2_, γ-Al_2_O_3,_ and sillimanite (Al_2_O_3_•SiO_2_). The low reactivity of γ-Al_2_O_3_ and sillimanite (Al_2_O_3_•SiO_2_) resulted in the poor dissolution of Al_2_O_3_ and a decrease in n(Si/Al) [47]. Therefore, the roasting temperature was determined to be 650 °C.

Figure 1b reflects the effect of the hydrochloric acid concentration on n(Si/Al) under a roasting temperature of 650 °C, an acid leaching temperature of 160 °C, and an acid leaching time of 6 h. The analysis in Figure 1b shows that with the increase in the hydrochloric acid concentration, the n(Si/Al) of the acid leaching sample showed a trend of first increasing and then stabilizing. When the concentration of hydrochloric acid was 2–6 mol/L, the Al_2_O_3_ content of the acid-leached sample gradually decreased to 5.32%, the SiO_2_ content increased to 81.76%, and the n(Si/Al) of the acid-leached sample reached 26.14. This was because the increase in hydrochloric acid concentration increased the H^+^ concentration of the reaction system; it also increased the reaction activity, Al_2_O_3_ dissolution rate, and n(Si/Al) of the acid-leached sample. When the concentration of hydrochloric acid was higher than 6 mol/L, the n(Si/Al) of the acid-leached sample tended to stabilize. Therefore, the concentration of hydrochloric acid is determined to be 6 mol/L.

Figure 1c reflects the effect of the acid leaching temperature on n(Si/Al) under a roasting temperature of 650 °C, a hydrochloric acid concentration of 6 mol/L, and an acid leaching time of 6 h. The analysis in Figure 1c shows that as the acid leaching temperature increased, the n(Si/Al) of the acid leaching sample showed a trend of first increasing and then stabilizing. When the acid leaching temperature was 120–160 °C, the n(Si/Al) of the acid slag increased from 13.17 to 26.14, which was because the increase in reaction temperature was conducive to an improvement in the diffusion rate and effective collision probability of the molecules [48]. When the acid leaching reached a certain temperature, there was autogenous pressure in the reactor. The higher the acid leaching temperature, the greater the pressure; this promoted the entry of H^+^ into the pores of the coal gangue, improving the diffusion ability of H^+^ in the interior of the coal gangue and making it easier for the active components inside the coal gangue to be dissolved. The acid leaching temperature continues to rise, and the n(Si/Al) of the acid leaching sample tended to stabilize, indicating that the aluminum-containing minerals (except quartz and feldspar) had dissolved at 160 °C. Therefore, the acid leaching temperature was determined to be 160 °C.

Figure 1d reflects the effect of the acid leaching time on n(Si/Al) under a roasting temperature of 650 °C, a hydrochloric acid concentration of 6 mol/L, and an acid leaching temperature of 160 °C. The analysis in Figure 1d shows that with the increase in the acid leaching time, the n(Si/Al) of the acid leaching sample showed a trend of first increasing and then stabilizing. When the acid leaching time was 2–6 h, the n(Si/Al) increased from 15.67 to 26.14, and the dissolution rates of Al_2_O_3_ and Fe_2_O_3_ were faster. This was because at the initial stage of the reaction, the concentration of hydrochloric acid was high, the reaction was intense, and the reaction rate was fast. When the acid leaching time was longer than 6 h, the n(Si/Al) of the acid leaching sample tended to be flat. Therefore, the acid leaching time was determined to be 6 h.

The leaching ability of Al was enhanced by pressure acid leaching. Under the optimal conditions, the n(Si/Al) of coal gangue increased to 26.14, which reached the theoretical n(Si/Al) requirement for the preparation of ZSM-5 molecular sieve. To explore the impact of n(Si/Al) on the preparation of ZSM-5 molecular sieves from coal gangue, in the acid leaching samples, 4 samples with a gradient of 5 of n(Si/Al) were selected as raw materials for later experiments. The process parameters for different n(Si/Al) samples are shown in Table 1.

Table 1 shows the n(Si/Al) of the acid-leached samples under different acid leaching conditions. The highest n(Si/Al) was 26.14, and the Fe_2_O_3_ content in the coal gangue was reduced from 7.13% to 0.58%. The SiO_2_ content increased from 46.19% to 81.76%, achieving effective enrichment of silicon. The content of Al_2_O_3_ decreased from 12.60% to 5.32%, and the n(Si/Al) of the acid slag increased from 6.23 to 26.14 based on the calculation (Equation (1)). After consulting the literature, the minimum n(Si/Al) for preparing a ZSM-5 molecular sieve was determined to be 15 [44], which proved the theoretical feasibility of preparing a ZSM-5 molecular sieve using onlt coal gangue as the silicon and aluminum source. n(Si/Al) has a significant impact on the preparation of ZSM-5 molecular sieves. Different n(Si/Al) directly lead to the crystallinity, morphology, and pore size characteristics of ZSM-5 molecular sieves [49,50]. The influence of n(Si/Al) on the preparation of ZSM-5 molecular sieves was investigated using acid leaching samples with different n(Si/Al) as raw materials.

### 3.2. Effect of n(Si/Al) on the Preparation of ZSM-5 Molecular Sieve

**Figure 2:**
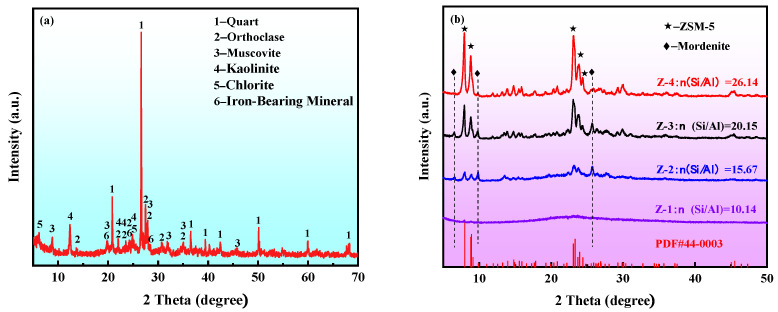
XRD of the samples: (**a**) coal gangue; (**b**) ZSM-5.

**Figure 3:**
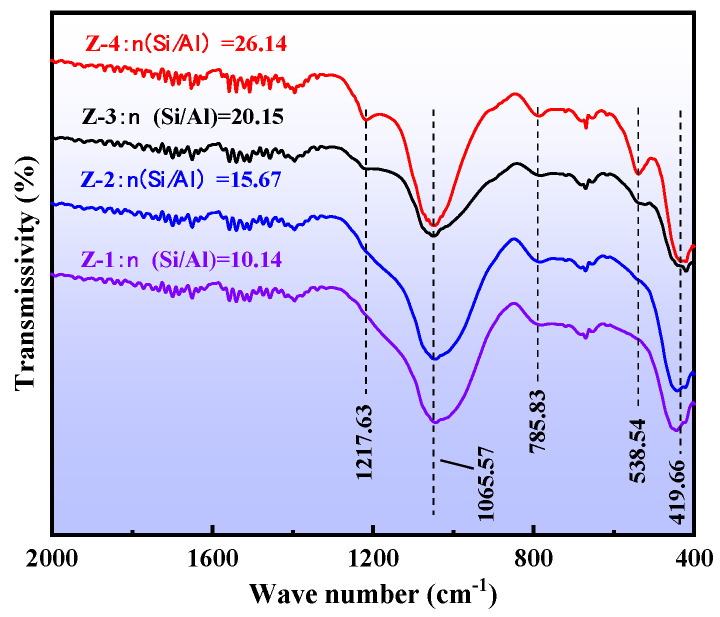
Infrared spectrogram of ZSM-5.

**Figure 4:**
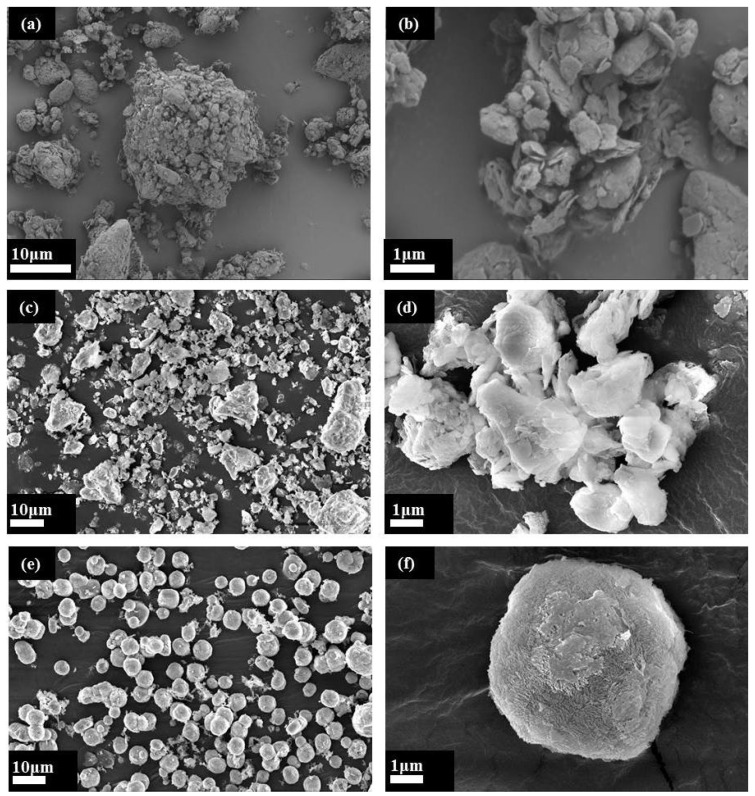
SEM morphology analysis: (**a**,**b**) coal gangue SEM; (**c**,**d**) acid leaching sample SEM; (**e**,**f**) ZSM-5 SEM.

**Figure 5:**
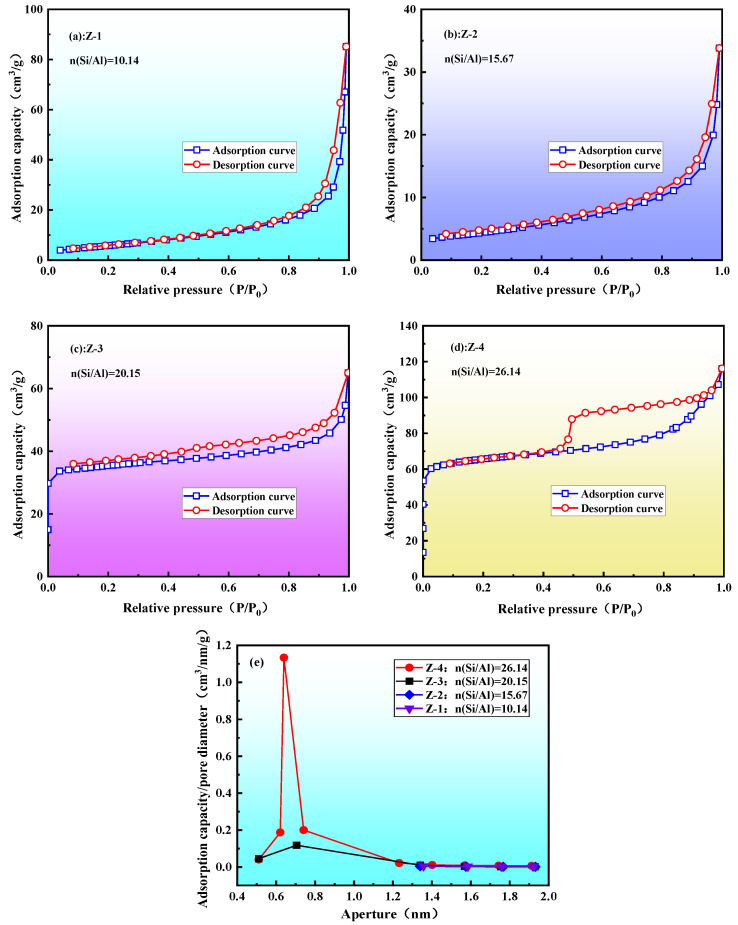
Analysis of pore structure of ZSM-5 molecular sieve: (**a**–**d**) N_2_ adsorption–desorption isotherms of ZSM-5; (**e**) pore-size distribution of ZSM-5.

Due to the stable structure of quartz and feldspar, they exhibited no reactive activity with hydrochloric acid after the medium-temperature chlorination roasting. It was, therefore, necessary to destroy their mineral structure by alkali melting to obtain an active silicon and aluminum source, and then prepare the ZSM-5 molecular sieve using the hydrothermal method. The XRD (Figure 2), FT-IR (Figure 3), SEM (Figure 4), and pore structure (Figure 5, Table 2) of the ZSM-5 molecular sieve samples (Z-1, Z-2, Z-3, and Z-4) were analyzed; these had been prepared at *n* = (Si/Al) (*n* = 10.14, 15.67, 20.15, and 26.14). The ratio was SiO_2_:xAl_2_O_3_:0.04TPABr:140H_2_O, the ZSM-5 seed dosage was 0.0018 g, the pH = 10.5, the crystallization temperature was 180 °C, and the crystallization time was 24 h.

#### 3.2.1. XRD Analysis of the ZSM-5 Molecular Sieve

The analysis in Figure 2a shows that the characteristic diffraction peak of quartz appeared in the spectrum, indicating that the Si in the coal gangue mainly existed in the form of crystalline quartz. There were also obvious characteristic diffraction peaks (such as kaolinite, feldspar, mica, and chlorite) on the map. These all belonged to aluminosilicate minerals, indicating that Al and some Si in the coal gangue existed in the form of aluminosilicate minerals. Figure 2b shows that with the increase in n(Si/Al), the characteristic diffraction peak intensity of the prepared ZSM-5 molecular sieve was higher, and there was a gradual reduction in miscellaneous peaks. When n(Si/Al) = 10.14, the sample presented a dispersion peak at 20~30°, indicating that no ZSM-5 crystals were produced. When n(Si/Al) = 15.67, weak characteristic diffraction peaks of ZSM-5 could be seen at 7.972°, 8.818°, 8.917°, 23.079°, 23.319°, and 23.949° in the spectrum. Although the minimum n(Si/Al) required for the synthesis of ZSM-5 molecular sieves was reached, the crystallization was incomplete. Therefore, it was verified that the lower limit of n(Si/Al) for the preparation of the ZSM-5 molecular sieve was 15. When n(Si/Al) = 20.15, the characteristic diffraction peak of ZSM-5 could be seen, but it was also accompanied by the crystalline phase of mordenite, and the relative crystallinity was poor. When n(Si/Al) = 26.14, the pattern showed a typical MFI topological structure diffraction peak, and there was no impurity peak. The relative crystallinity of the sample was the highest, indicating that a pure-phase ZSM-5 molecular sieve had been prepared. According to the Lowenstein rule, the two aluminum atoms on the tetrahedral position of the molecular sieve skeleton cannot be adjacent. Therefore, higher n(Si/Al) in the crystallization synthesis system can promote the formation of the crystal nucleus of a ZSM-5 molecular sieve. Figure 2b shows that when n(Si/Al) was 26.14, the characteristic diffraction peaks of quartz and kaolinite did not appear in the XRD pattern. This indicated that the crystal structure of the coal gangue mineral had been destroyed, and a ZSM-5 molecular sieve with coal gangue as the silicon and aluminum source had successfully been prepared.

#### 3.2.2. FT-IR Analysis of the ZSM-5 Molecular Sieve

The analysis in Figure 3 shows that, according to the FKS rule, the infrared absorption peak at 1065.57 cm^−1^ belonged to the asymmetric stretching vibration of the T-O-T (T represents Si or Al) bond, the infrared absorption peak at 785.83 cm^−1^ belonged to the symmetric stretching vibration of the T-O bond, and the infrared absorption peak at 419.66 cm^−1^ belonged to the variable angle vibration absorption peak of T-O, indicating that the absorption peaks at 1065.57 cm^−1^, 785.83 cm^−1^, and 419.66 cm^−1^ all belonged to the internal vibration of the Si(Al)O_4_ tetrahedron. The absorption peak at 1217.63 cm^−1^ was attributed to the asymmetric contraction vibration of the outer tetrahedron of T-O-T [51]. The absorption peak at 538.54 cm^−1^ belonged to the double-pentacyclic vibration of the skeleton, which was a typical characteristic absorption peak of a ZSM-5 molecular sieve [52]. The simultaneous presence of absorption peaks at 1217.63 cm^−1^, 1065.57 cm^−1^, 785.83 cm^−1^, 538.54 cm^−1^, and 419.66 cm^−1^ indicated that ZSM-5 molecular sieves had successfully been prepared. When n(Si/Al) was between 10.14 and 15.67, there was no absorption peak at 538.54 cm^−1^ in the spectrum; this region belongs to the vibration absorption peak of the double-pentacyclic framework of the ZSM-5 molecular sieve. When n(Si/Al) was 20.15, the infrared absorption peak was obvious at 538.54 cm^−1^, indicating that many ZSM-5 molecular sieve skeletons had been formed. When n(Si/Al)) was 26.14, the absorption peak intensity is the highest, and the prepared ZSM-5 molecular sieve skeleton structure was the best. In the XRD analysis results, when n(Si/Al)) was 26.14, the characteristic peak of the prepared ZSM-5 molecular sieve sample was the strongest. The two characterization methods fully indicated the successful preparation of ZSM-5 molecular sieve materials.

#### 3.2.3. Morphological Analysis of the ZSM-5 Molecular Sieve

Figure 4a shows that the coal gangue presented irregular clumps or floccules, with uneven sizes. Figure 4b shows that the surface of the coal gangue was relatively smooth, indicating that the clumps and floccules in the coal gangue were formed by stacking of the flaky structure, with a small distance between the layers. There was a certain number of narrow pores within the nanometer range. A comparative analysis between Figure 4a,c showed that after the pressure acid leaching treatment, the coal gangue opened into irregular clumps or flocs; the image is mainly composed of quartz and feldspar particles. A comparative analysis between Figure 4b,d showed that the surface of the flaky particles was rough, which was due to the removal of clay minerals, such as kaolinite from the coal gangue via pressurized acid leaching, resulting in defects and voids on the surface. Figure 4e shows that the prepared ZSM-5 molecular sieve was a spherical particle with less amorphous material and high sample purity. As shown in Figure 4b, the spherical particles were crystal clusters formed by the aggregation of ZSM-5 crystals. The formation of clusters was due to the addition of ZSM-5 seeds, which caused the primary crystal nucleus to grow into spherical clusters at the defects of the ZSM-5 seeds. The strong ordering of the grains and the existence of gaps between the grains within the nanometer range indicate the existence of intergranular mesopores in the sample.

#### 3.2.4. Pore Structure Analysis of ZSM-5 Molecular Sieve

In Figure 5a,b, when n(Si/Al) = 10.14–15.67, the N_2_ adsorption–desorption isotherm of the sample conformed to a type III isotherm curve, with a weak force between the sample and the nitrogen molecule, and a small amount of adsorption in the low-pressure region [53]. Table 2 (Z-1, and Z-2) shows that the specific surface area of the micropores was 0 cm^2^/g, indicating that there was no formation of ZSM-5 micropores, mainly amorphous silicon–aluminum active substances. This was due to the high aluminum content, which hindered the formation of ZSM-5 double five-membered rings, leading to crystallization failure. Figure 5c,d shows that when n(Si/Al) = 20.15–26.14, the N_2_ adsorption–desorption isotherm of the sample conformed to a type-I isotherm in the low-pressure region, which was a typical microporous adsorption isotherm. When n(Si/Al) = 26.14, the maximum adsorption amount in the low-pressure region was 60 cm^3^/g, indicating that the higher the n(Si/Al), the greater the benefit to the formation of ZSM-5 molecular sieve crystals and the greater the microporous channels. A hysteresis loop appeared at a relative pressure of 0.4 < P/P0 < 1 in the N_2_ adsorption–desorption curve. Combined with the SEM analysis results in Figure 4f, we observed that the spherical particles were crystal clusters formed by the aggregation of ZSM-5 crystals. This indicated that there were intercrystalline mesopores in the sample, resulting in the hysteresis of the N_2_ adsorption–desorption curve; the pore type was a slit pore. The T-Plot results in Table 2 of Z-4 show a specific surface area of 169.6329 m^2^/g, an H-K pore volume of 0.0988 cm^3^/g, and an average pore diameter of 0.6285 nm. In Table 2, there are comparisons with the ZSM-5 molecular sieves prepared with existing chemical reagents. Through further research and optimization, it is expected to realize the preparation of a ZSM-5 molecular sieve with coal gangue instead of chemical reagents.

**Table 2:** Pore structure datasheet of ZSM-5 prepared at different n(Si/Al).

Sample Number	Specific Surface Area (m^2^/g)		Aperture (nm)	Pore Volume (cm^3^/g)
	T-Plot	BET	H-K	H-K
Coal gangue	3.0519	20.5090	1.2798	0.0076
Z-1	0.0000	21.6663	—	—
Z-2	0.0000	15.2170	—	—
Z-3	90.9119	106.4974	0.5413	0.0535
Z-4	169.6329	203.6859	0.6285	0.0988
ZSM-5 [54]	195.2600	407.8100	—	0.1003
HZSM-5 [55]	81.4100	137.8000	—	0.0400

## 4. Conclusions

In this study, ZSM-5 molecular sieves were prepared using the hydrothermal method, with coal gangue as the raw material and hydrochloric acid as the acid leaching medium. Medium-temperature chlorination roasting and the pressure acid leaching process were used to control the n(Si/Al) of the coal gangue. The effect of n(Si/Al) on the preparation of ZSM-5 molecular sieves from coal gangue was studied. The conclusions was as follows:

(1) Medium-temperature chlorination roasting and the pressure acid leaching process were used to effectively remove elements, such as Fe and Al, from coal gangue, increasing the n(Si/Al) of the coal gangue from 6.13 to 10.14, 15.67, 20.15, and 26.14. The pressure acid leaching process solved the limitation that mica and kaolinite in coal gangue cannot be simultaneously activated. Under optimal conditions, the n(Si/Al) of the coal gangue increased to 26.14, which met the requirements for the preparation of a ZSM-5 molecular sieve.
(2) Using only coal gangue as the silicon and aluminum source, a ZSM-5 molecular sieve was successfully prepared. The minimum n(Si/Al) for the preparation was determined to be 20.15. The microstructure of the ZSM-5 molecular sieve prepared under optimal conditions showed spherical particles, which were formed by the aggregation of ZSM-5 grains. The sample had a microporous specific surface area of 169.6329 m^2^/g, an average pore diameter of 0.6285 nm, and a pore volume of 0.0988 cm^3^/g.

## Figures and Tables

**Table 1:** The n(Si/Al) control process datasheet.

Sample Number	Acid Leaching Process Parameters				n(Si/Al)
	Roasting Temperature (°C)	HCl Concentration (mol/L)	Acid Leaching Temperature (°C)	Acid Leaching Time (h)	
1	850	8	160	6	10.14
2	650	6	160	2	15.67
3	650	6	160	4	20.15
4	650	6	160	6	26.14

## Data Availability

Not applicable.
